# Anticoagulation Patterns in Ischemic Stroke Patients with Atrial Fibrillation in Developing Country: Insights from the Stroke Registry in Vietnam

**DOI:** 10.3390/jcdd11090269

**Published:** 2024-08-30

**Authors:** Mai Duy Ton, Dao Viet Phuong, Nguyen Tien Dung, Nguyen Van Chi, Truong Thi Hoa, Tran Cong Minh, Jeyaraj Pandian, Nguyen Huy Thang

**Affiliations:** 1 Stroke Center, Bach Mai Hospital, Hanoi 100000, Vietnam; tonresident@gmail.com (M.D.T.); daovietphuong85@gmail.com (D.V.P.); nguyentiendungtm29@gmail.com (N.T.D.); 2 Faculty of Stroke and Cerebrovascular Disease, VNU-University of Medicine and Pharmacy, Hanoi 100000, Vietnam; truonghoahmu@gmail.com; 3 Department of Emergency and Critical Care Medicine, Hanoi Medical University, Hanoi 100000, Vietnam; chinvvn@yahoo.com; 4 Emergency Center, Bach Mai Hospital, Hanoi 100000, Vietnam; 5 Nuffield Department of Clinical Neuroscience, University of Oxford, Oxford OX1 2JD, UK; minh.tran@ndcn.ox.ac.uk; 6 Christian Medical College, Ludhiana 141001, Punjab, India; jeyaraj.pandian@cmcludhiana.in; 7 Department of Cerebrovascular Disease, The People’s Hospital 115, Ho Chi Minh City 700000, Vietnam; 8 Department of Neurology, Pham Ngoc Thach University of Medicine, Ho Chi Minh City 700000, Vietnam

**Keywords:** atrial fibrillation, ischemic stroke, oral anticoagulation, Vietnam, RES-Q, stroke prevention

## Abstract

(1) Background: Atrial fibrillation (AF) poses a growing cardiovascular challenge globally, with significant implications in Vietnam. This study aimed to investigate the impact of AF on ischemic stroke/transient ischemic attack (TIA) and clinical outcomes, as well as the utilization of oral anticoagulation (OAC) therapy in Vietnam. (2) Methods: Data from the Registry of Stroke Care Quality (RES-Q) across 66 hospitals in Vietnam between 2017 and 2023 were utilized. Patients diagnosed with ischemic stroke or TIA were included, and clinical characteristics, pre- and post-hospitalization medication, and hospitalized outcomes were examined. (3) Results: Of 94,144 patients, 15.1% had AF. Patients with AF were older and had a higher prevalence of heart failure and more severe strokes. AF was associated with increased mortality during hospitalization and a poorer prognosis. In AF patients, anticoagulation therapy utilization increased from 15.8% pre-hospitalization to 82.4% at discharge, with a preference for direct oral anticoagulants. (4) Conclusions: AF significantly impacts ischemic stroke/TIA outcomes in Vietnam. Pre-hospitalization and anticoagulation therapy utilization were low but improved at discharge. These findings emphasize the need for improved AF management and stroke prevention strategies in developing countries.

## 1. Introduction

Atrial fibrillation (AF) represents a significant and growing cardiovascular challenge with global implications, including a notable impact in Vietnam. Current epidemiological data suggest that AF prevalence in the Vietnamese general population approximates 1%, with a progressive increase in incidence correlating with advancing age—evidenced by a prevalence of 3.9% in individuals aged 60 years and above, and 9% in those aged 80 years and older [1,2]. Given the demographic transition towards an ageing population, it is projected that AF will impose an escalating burden on the healthcare system in Vietnam [3]. Additionally, AF is a critical etiological factor, accounting for 18.1% of ischemic stroke and transient ischemic attack (TIA) cases in Vietnam [4]. Strokes of cardioembolic origin are associated with a comparatively poorer prognosis than strokes from other causes [5].

Anticoagulation therapy is the prophylactic mainstay for stroke prevention in patients with AF. Notwithstanding numerous initiatives aimed at refining the care for atrial fibrillation, a persistent issue in the management of this condition within developing countries, including Vietnam, is the suboptimal utilization of anticoagulation [1,2,6]. This encompasses both the non-utilization of anticoagulants when indicated and the administration of dosages that deviate from established evidence-based guidelines. The clinical implications of this underutilization are profound, given the well-established association between the insufficient use of anticoagulation and adverse clinical outcomes in AF patients. Therefore, insights into anticoagulation practices for AF, derived from large databases, are crucial for identifying treatment gaps and devising appropriate corrective measures.

To support the efforts toward stroke management implementation in Vietnam, the Registry of Stroke Care Quality (RES-Q) was deployed. RES-Q is an instrumental platform aimed at enhancing global stroke care by identifying gaps and promoting the implementation of evidence-based practices among healthcare professionals and policymakers. In Vietnam, RES-Q was initiated in 2017. During the past seven years and to date, 48 stroke centres have enrolled in the program [7]. This is one of the major efforts to improve stroke care quality in Vietnam.

In this study, we aimed to study the ischemic stroke/TIA attributable to AF and clinical outcomes in patients with and without AF in Vietnam. We also studied the use of OAC therapy in patients with AF. 

## 2. Methodology

### 2.1. Study Design and Patient Inclusion

Patients were selected from six cohorts spanning from 1 June 2017 to 31 December 2023, using data obtained from the RES-Q across 66 hospitals in Vietnam. The hospitals included in RES-Q are provincial-level facilities situated across the three regions of North, Central, and South Vietnam, as well as in the central cities of Ho Chi Minh City and Hanoi. These designated hospitals are required to have a dedicated stroke centre or unit. 

**Figure 1:**
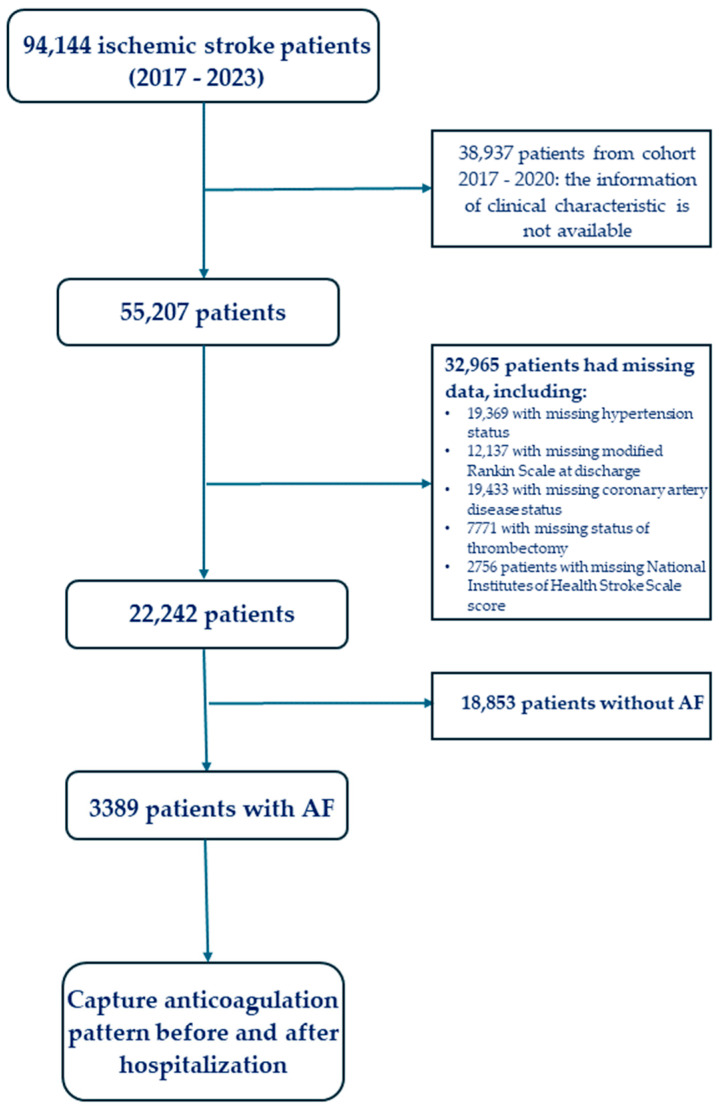
Diagram of patient selection.

Eligible patients comprised those diagnosed with ischemic stroke or TIA. Stroke and TIA in RES-Q are diagnosed based on clinical symptoms presenting with focal neurological signs and brain imaging, in accordance with the criteria outlined by the American Heart Association (AHA) [8]. Subsequently, these patients undergo evaluation for the mechanism of ischemic stroke, following the TOAST classification as recommended by the AHA [9]. Data collection focused on clinical characteristics, medication before and after hospital admission, and clinical outcomes during hospitalization. The flowchart depicting patient selection is illustrated in Figure 1. Exclusion criteria encompassed patients presenting with intracranial hemorrhage, cerebral venous thrombosis, stroke mimics, subarachnoid hemorrhage, and undetermined stroke. 

### 2.2. Data Collection

Data collection in the RES-Q database begins with users registering an account and inputting patient data from medical records. These data are stored securely within a cloud IT infrastructure in the EU, adhering to the Czech Republic and the EU’s GDPR legislation. Access to personal data is strictly limited to authorized users managing RES-Q. The platform employs stringent security measures to prevent unauthorized access or data modification, with all data transmitted securely using Transport Layer Security (TLS) over HTTP (HTTPS), ensuring the integrity and confidentiality of patient data.

The management of the RES-Q database is overseen by the Health Management Institute (HMI), a non-profit organization based in Brno, Czech Republic. Access to the national source or aggregated data within RES-Q for scientific, academic research, or quality improvement purposes can be obtained by approaching the RES-Q National Coordinator (NC), which serves as the main point of contact between institutions registered in RES-Q and the HMI. 

### 2.3. Ethical Statement

In accordance with ethical standards, all data within the RES-Q database are de-identified to protect patient confidentiality. Patient data are uploaded using a pseudonymized ID, devoid of any information that could link the data to an identifiable individual. RES-Q has the capability to automatically generate these pseudonymized IDs. Alternatively, participating institutions may opt to employ their pseudonymization schemes.

### 2.4. Statistical Analysis

Patients with incomplete datasets were excluded from the analysis. Subsequent to data cleansing, Stata version 17.0 was employed for statistical analysis. Mean values and standard deviations were computed for continuous variables such as age, NIHSS scores, and mRS scores. To ascertain the statistical significance of differences between groups, Student’s t-tests were utilized for continuous variables while chi-square tests were applied to categorical variables. A *p*-value threshold of less than 0.05 was established for determining statistical significance.

## 3. Results

### 3.1. Baseline Patient Characteristics

During the period extending from 2017 to 2023, a cohort of 94,144 patients with a diagnosis of ischemic stroke or TIA were enrolled in the RES-Q database from Vietnam. Amongst these patients, 14,185 individuals were either known to have a history of AF or were diagnosed with AF at the time of admission, constituting 15.1% of the total cohort. Among the cases of AF, 2389 patients were diagnosed with atrial fibrillation during hospitalization (70.5%), while 1000 patients (29.5%) had a known history of atrial fibrillation. It is imperative to acknowledge that comprehensive clinical characteristics of patients are exclusively available and complete for the years spanning 2021 to 2023. In the 2021–2023 cohort, 32,540 patients (58.94%) had incomplete datasets. A significant proportion of the data from 2021, encompassing baseline characteristics and treatment information, is absent. The patient cohort with AF exhibited a higher mean age compared to those without AF (69.5 ± 12.4 years vs. 66.3 ± 12.7 years; *p* < 0.001). The prevalence of risk factors and comorbid conditions, such as hypertension, diabetes, and smoking, appeared more frequently in the non-AF cohort. Notably, the incidence of heart failure was significantly greater in patients with AF (8.5% vs. 1.0% in the non-AF group; *p* < 0.001). Among patients with AF, the utilization of anticoagulant therapy before hospitalization was observed in a mere 15.8%, while 11.1% of patients were administered antiplatelet agents. Additionally, the severity of ischemic stroke in individuals with AF was notably greater compared to those without the condition, as evidenced by a significantly elevated mean NIHSS score in the AF cohort (10.8 ± 5.9 vs. 7.0 ± 5.5, *p* < 0.001). The clinical characteristics of the AF cohorts and non-AF cohorts are detailed in Table 1.

### 3.2. Impact of Atrial Fibrillation on Mortality and Severity of Ischemic Stroke

**Figure 2:**
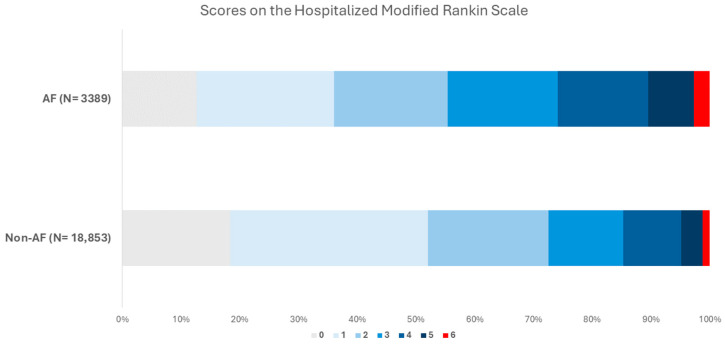
The hospitalized clinical outcome.

Patients with ischemic stroke/TIA without AF exhibited a lower incidence of poor outcomes, as defined by a mRS score of 3–6, compared to patients with AF (OR 0.58, 95% CI 0.50–0.68, *p* < 0.01). Additionally, the mortality risk was lower in patients without AF than in patients with AF (OR 0.31, 95% CI 0.22–0.43, *p* < 0.01). The distribution of patients stratified by mRS is presented in Figure 2. The incidence of severe stroke, as defined by a Modified Rankin Scale (mRS) score of 3–5, was significantly elevated in the cohort with AF. Prognostic evaluations revealed a more adverse long-term outcome for strokes associated with AF, as evidenced by a higher median mRS (IQR) in patients with AF of 2 (1–4) compared to those without AF, with a median mRS of 1 (1–3), *p* < 0.05. 

### 3.3. Treatment Pattern

**Figure 3:**
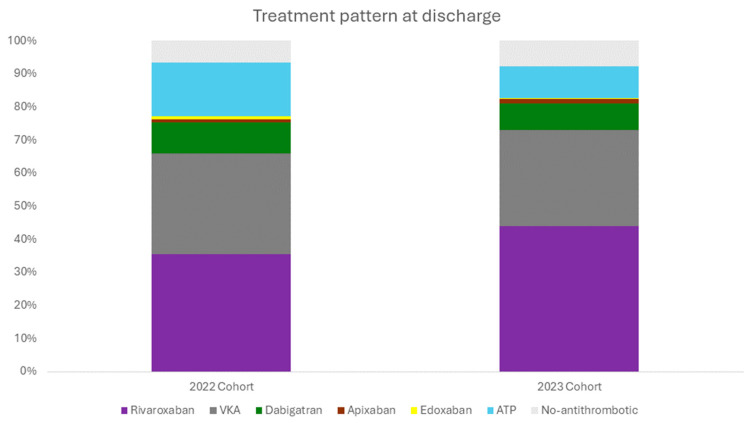
Treatment pattern of AF at hospital discharge; 2022–2023.

In patients with ischemic stroke or TIA attributed to atrial fibrillation, the utilization of anticoagulants prior to the event was notably low, with only 15.8% of patients using anticoagulants. Additionally, 11.1% of patients were found to have used antiplatelets. Among stroke survivors, 82.4% were administered anticoagulation therapy at the time of discharge. The cohort not receiving anticoagulation therapy at discharge was characterized by an older mean age (70.9 ± 12.1 years) compared with those who were prescribed anticoagulation (69.2 ± 12.5 years; *p* < 0.001). Additionally, this group exhibited a higher baseline NIHSS score (12.5 ± 7.0 versus 10.5 ± 5.5; *p* < 0.001) and an increased mean mRS score (3.1 ± 1.9 versus 2.2 ± 1.5, *p* < 0.001). The clinical characteristics of OAC-receiving patients and non-OAC-receiving patients are detailed in Table 2. The utilization of anticoagulation therapy in stroke survivors increased between 2022 and 2023 (77.2% OAC 2022 vs. 82.7% OAC 2023, *p* < 0.03). Rivaroxaban was the most frequently prescribed anticoagulant (50.1%), followed by vitamin K antagonists (37.0%) and dabigatran (10.6%), while apixaban and edoxaban were prescribed to a lesser extent. The prescription rate of antiplatelet agents declined over the same period (16.2% in 2022 vs. 9.5% in 2023, *p* < 0.001). Figure 3 illustrates the specific treatment pattern in detail.

## 4. Discussion

The findings from our study, encompassing a large cohort of stroke patients over an extended period from 2017 to 2023, indicate that AF is an important etiology of acute ischemic stroke in Vietnam, representing 15.1% of cases. This aligns with prior research within the Vietnamese context, which reported AF in 18.1% of individuals experiencing ischemic stroke, or TIA [4]. Consistent with an extensive body of the previously published literature, our study reveals that Vietnamese patients who experience stroke attributable to AF have an increased hospital mortality rate and an overall poorer prognosis when compared to patients without AF [10]. The prevalence data, coupled with the observed clinical outcomes, convey a clear message: AF represents a substantial challenge to the healthcare system in Vietnam, as well as in other developing nations, with significant implications for public health and resource allocation. It is critical to acknowledge that in the RES-Q database, AF screening was predominantly conducted using a 12-lead electrocardiogram, which may not detect paroxysmal AF, leading to potential underdiagnosis. Intensive screening for AF in all patients presenting with acute stroke may not be practical in Vietnam or similar resource-constrained settings given the associated costs and the burden on the healthcare infrastructure. A targeted approach might involve prioritizing high-risk patient populations for atrial fibrillation screening during routine evaluations at primary care facilities. Additionally, the deployment of smart digital devices for arrhythmia detection in high-risk cohorts could be a viable strategy. However, further investigation is warranted to determine the cost-effectiveness of such interventions for the healthcare system and the patient population.

The results of our investigation present encouraging developments in the management of cardiovascular conditions, such as hypertension and dyslipidemia, within the developing healthcare setting. Hypertension and dyslipidemia are well-documented risk factors for stroke, and their management through antihypertensive drugs and statins, respectively, is associated with a reduction in stroke risk [11,12]. In our cohort, upon admission, 16,317 patients presented with hypertension, of whom 14,413 were receiving antihypertensive medication. Additionally, 2876 patients had dyslipidemia, with 2210 of them receiving statin therapy. These findings suggest a growing awareness among healthcare professionals (HCPs) and patients in developing nations of the importance of controlling these conditions. Conversely, the utilization of anticoagulants for stroke prevention in patients with AF presents a more concerning scenario. Only 15.8% of patients with AF were prescribed anticoagulants, and 11.1% were on antiplatelets, with a substantial majority not receiving any form of antithrombotic therapy. This rate is considerably lower than that observed in developed countries, where 66.6–79.3% of patients with AF are treated with anticoagulants [13,14]. Oral anticoagulation remains the cornerstone for the prevention of AF-associated stroke, concurrently reducing the incidence of stroke and mortality [15,16]. The underuse of anticoagulation is primarily attributed to inadequate awareness among HCPs and patients regarding AF and the benefits of anticoagulant therapy, as well as apprehensions concerning bleeding risks and misconceptions about the safety of anticoagulants [1]. There is a pressing need for more comprehensive medical education initiatives targeting both HCPs and the general public in developing countries, including Vietnam, to enhance understanding of AF and the significance of anticoagulation therapy. Moreover, there is a call for physicians to broaden their perspective on the safety of anticoagulant use. Safety should not be solely associated with the risk of bleeding; rather, it should be conceptualized as the prevention of more detrimental events that could impact patient outcomes.

The landscape of secondary stroke prevention among patients with AF in Vietnam is showing encouraging trends, with 82.4% of patients being prescribed anticoagulation therapy upon hospital discharge. This figure is similar to that in developed countries, where the prescription rate ranges from 69.5 to 71.0% [17,18]. A notable disparity exists between secondary and primary prevention, potentially due to the tangible consequences of stroke secondary to AF, which may enhance the perceived necessity of prophylactic measures among both clinicians and patients. In the realm of anticoagulant selection for secondary stroke prevention, the Vietnamese patient population predominantly opts for direct oral anticoagulants (DOACs), with rivaroxaban being the most administered. Between 2022 and 2023, there has been a marked increase in the utilization of anticoagulants and DOACs. The broader clinical integration of DOACs has been associated with improved clinical outcomes in patients with atrial fibrillation, underscoring the positive impact of concerted efforts to optimize acute stroke care in developing countries, including Vietnam. Patients who were not prescribed anticoagulation for secondary prevention exhibited higher baseline NIHSS and mRS scores compared to those who received anticoagulation, suggesting that the decision against prophylactic anticoagulation may be influenced by a projected poorer prognosis in this subgroup, potentially leading to a devaluation of its preventive benefits. Additionally, a significant percentage of patients with AF (12.5%) are still discharged with antiplatelets, indicating that there is substantial room for improvement in the management of secondary AF-related stroke. Nonetheless, it is imperative to reiterate the principle that ‘earlier is better’ in the context of AF-related stroke prevention. 

Despite the considerable scale of this study and the valuable insights it offers into the management of atrial fibrillation among the ischemic stroke/TIA population in Vietnam, it is imperative to consider the limitations inherent in our research. Firstly, a substantial portion of the patients included in the registry data had incomplete records. This issue may stem from the high patient volumes at major stroke centres in previous years, which could impede the comprehensive assessment and documentation of individual patient data. To mitigate the potential impact on our findings, we excluded all patients with one or more missing data points. Secondly, while the RES-Q database effectively captured patterns of anticoagulation use, it unfortunately did not include data on treatment adherence, a critical aspect of evaluating the efficacy of treatment. Assessing the quality of care requires not only an examination of prescriptive practices, which reflect healthcare provider awareness, but also patient compliance, a key indicator of patient engagement with their treatment regimen. Comprehensive data encompassing both of these facets would enable more targeted and precise interventions. Thirdly, this dataset was limited to in-hospital observations, with no follow-up data available to assess long-term outcomes such as 90-day mortality, bleeding events associated with anticoagulation therapy, or rates of stroke recurrence. Lastly, as with any retrospective analysis, our study is subject to the limitations commonly associated with such research, including potential variability in diagnosis, assessment, and intervention across the different centres contributing to the RES-Q database.

## 5. Conclusions

In conclusion, the present study underscores the significance of AF as a contributory factor to ischemic stroke and TIA. The association of AF with an elevated risk of mortality during hospitalization and a poorer prognosis in the ischemic stroke/TIA cohort is noteworthy. Our findings highlight stark differences in the approach to stroke prevention pre- and post-hospitalization for stroke. Before hospital admission for stroke, the utilization of anticoagulation therapy was notably low, with only 15.8% of patients on such treatment. However, this figure shows a considerable increase upon discharge, with 82.4% of patients being prescribed anticoagulants.

## Figures and Tables

**Table 1:** Baseline patient characteristics and results of Student’s t-test in comparison of the non-atrial fibrillation and atrial fibrillation. TIA is the transient ischemic attack, MI is myocardial infarction, rTPA is the recombinant tissue plasminogen activator, NIHSS is the National Institutes of Health Stroke Scale, and SD is the standard deviation.

Characteristics	Patients Cohort (2021–2023)		*p*-Value
	Non-AF Cohort (n = 18,853)	AF Cohort (n = 3389)	
Male, n (%)	11,371 (60.3)	1899 (56.0)	<0.001
Age, mean (SD), years	66.3 (12.7)	69.5 (12.4)	<0.001
Hypertension, n (%)	13,952 (74.0)	2419 (71.4)	<0.001
Diabetes, n (%)	5759 (30.5)	831 (24.5)	<0.001
Dyslipidemia, n (%)	2463 (13.1)	413 (12.2)	0.161
Smoking, n (%)	2916 (15.5)	335 (9.9)	<0.001
History of stroke/TIA, n (%)	2422 (12.8)	335 (9.9)	<0.001
Coronary artery disease/history of MI, n (%)	693 (3.7)	145 (4.3)	0.09
Congestive heart failure, n (%)	194 (1.0)	287 (8.5)	<0.001
Statin, n (%)	1897 (10.1)	313 (9.2)	0.139
Antihypertensive medication, n (%)	12,219 (64.8)	2194 (64.7)	0.935
Anticoagulant, n (%)	28 (0.1)	536 (15.8)	<0.001
Antiplatelet, n (%)	2896 (15.4)	378 (11.1)	0.472
rTPA, n (%)	2589 (13.7)	578 (17.1)	<0.001
Thrombectomy, n (%)	1690 (9.0)	762 (22.5)	<0.001
rTPA combine thrombectomy, n (%)	402 (2.1)	212 (6.3)	<0.001
NIHSS Median (IQR)	10 (7.0–14.0)	10 (6.0–14.8)	0.2

**Table 2:** Clinical characteristics of AF patients who received OAC and those who did not at hospital discharge.

Clinical Characteristics	AF Cohort (2021–2023)		*p*-Value
	Non-OAC (n = 598)	OAC (n = 2791)	
Male, n (%)	315 (52.7)	1584 (56.8)	0.068
Age, mean (SD), years	70.9 (12.1)	69.2 (12.5)	<0.001
Hypertension, n (%)	440 (73.6)	1979 (70.9)	0.190
Diabetes, n (%)	150 (25.1)	681 (24.4)	0.724
Dyslipidemia, n (%)	75 (12.5)	338 (12.1)	0.770
Smoking, n (%)	53 (8.9)	282 (10.1)	0.356
History of stroke/TIA, n (%)	68 (11.4)	267 (9.6)	0.180
Coronary artery disease/history of MI, n (%)	33 (5.5)	112 (4.0)	0.099
Congestive heart failure, n (%)	50 (8.4)	237 (8.5)	0.917
Statin, n (%)	325 (54.3)	1597 (57.2)	0.198
Antihypertensive medication, n (%)	398 (66.6)	2296 (82.3)	<0.001
rTPA, n (%)	104 (17.4)	474 (17.0)	0.810
Thrombectomy, n (%)	157 (26.3)	605 (21.7)	0.015
rTPA combine thrombectomy, n (%)	34 (5.7)	178 (6.4)	0.526
NIHSS Median (IQR)	12.0 (8.0–17.0)	10.0 (6.0–14.0)	<0.05
Modified Rankin Scale Median (IQR)	3.0 (2.0–5.0)	2.0 (1.0–3.0)	<0.05

## Data Availability

The corresponding author will provide the data upon reasonable request.
